# Editorial: Computational Approaches to Foster Innovation in the Treatment and Diagnosis of Infectious Diseases

**DOI:** 10.3389/fmedt.2022.841088

**Published:** 2022-02-04

**Authors:** Diana Lousa, Cláudio M. Soares, Fernando L. Barroso da Silva

**Affiliations:** ^1^Instituto de Tecnologia Química e Biológica António Xavier, Universidade Nova de Lisboa, Oeiras, Portugal; ^2^Departamento de Ciências Biomoleculares, Faculdade de Ciências Farmacêuticas de Ribeirão Preto, Universidade de São Paulo, Ribeirão Preto, Brazil; ^3^Department of Chemical and Biomolecular Engineering, North Carolina State University, Raleigh, NC, United States

**Keywords:** biomolecular interaction, bioinformatics, antibody development, SARS-CoV-2, infectivity

Infectious diseases are a continuous threat to human beings and *per se* a transdisciplinary and challenging subject. Joining efforts from experts coming from complementary research areas is the most effective way to provide a deeper understanding to diagnose, treat and prevent pathogenic threats. Computational approaches applied to the study of infectious diseases are creating a new research field that follows this multidisciplinary strategy, taking advantage of the great progress in molecular and structural biology, immunology, bioinformatics, and related areas, to foster the understanding of infectious diseases and drive innovation in their treatment, diagnosis, and prevention. From a fundamental description of viral and bacterial processes, and their molecular interactions in general, to the design of specific neutralizing antibodies, a diversity of clinical questions can now be addressed by computational methods. Such methods can benefit from the solid foundations that molecular simulation methods have achieved, together with the promises from machine learning techniques, to probe the thermodynamic, dynamic, and interactive properties of biomolecules with applications in material science, biotechnology, and health.

Characterization of the molecular aspects of viral and bacterial assembly, infectivity, and pathogenesis are intimately related with new alternatives to diagnosis, prevention, and treatment. The knowledge on other topics, such as virus evolutionary dynamics and cell/host-tropism are equally important for innovative strategies on drug design and discovery. When this Research Topic was initially planned, these Research Topics were especially timely due to the outbreaks of flaviviruses (especially Zika and Dengue). In the meantime, COVID19 arrived, shaping a new difficult reality and imposing a prompt response of science to deal with the ongoing COVID-19 pandemic. Huge efforts have been made to address all the aspects of SARS-CoV-2, which can be perceived by realizing the number of published papers on this subject. This Research Topic also reflects this scenario: all the accepted articles deal directly or indirectly with SARS-CoV-2.

A diversity of computational tools and molecular models at different levels can be seen in this Research Topic. Garay et al. presented a coarse-grained simulations' dataset of the SARS-CoV-2 proteome within the “The SIRAH-CoV-2 Initiative” framework. Such initiatives offered molecular dynamics (MD) trajectories that could allow any further analysis of large-scale dynamics of SARS-CoV-2 proteins. Such an idea was already explored in the work carried out by Giron et al. Using previously available MD coordinates as input in constant-pH Monte Carlo simulations, it was possible to theoretically predict the stability of the spike homotrimer for SARS-CoV-1 and different strains of SARS-CoV-2. It was found that the Beta variant does favor the open state conformation of the homotrimer which facilitates the virus-host cell attachment. CryoEM structures recently confirmed these predictions. Moreover, all studied variants in this theoretical work suggested an increased binding affinity between the receptor-binding domain and the angiotensin-converting enzyme II (ACE2). Alternative entering mechanisms for SARS-CoV-2 to access the human cells were further investigated by Bò et al. They evaluated the possibilities for SARS-CoV-2 to bind sialic acid molecules by extensive MD simulation using the CHARMM-27 force field.

Immunogenicity aspects were discussed by Nerli and Sgourakis employing the RosettaMHC Modeling Framework to predict and characterize T cell epitopes expressed by SARS-CoV-2. This is an important contribution to designing a new generation of vaccines for the prevention and therapeutic binders for treatments. As seen in the previous works mentioned above, this contribution also highlighted the importance of electrostatic interactions for virus mechanisms. In the same line, Boulard and Bressanelli discussed effective antiviral agents. They used a computational protocol to investigate the entering of cellular metabolites of Sofosbuvir through the NTP tunnel of hepatitis C virus NS5B.

Finally, Moreira et al. presented ViralFP: a web application of viral fusion proteins. Fusion proteins are key players in the entry process of enveloped viruses, such as HIV and influenza. The rise of COVID-19 showcased the importance of the SARS-CoV-2 spike fusion protein (the spike protein), which is the main therapeutic target of this virus and a hotspot for mutations found in the variants of concern. This application contains detailed information on known viral fusion proteins and provides a set of integrated tools, including a machine learning-based approach to predict the location of fusion peptides (the component of fusion proteins that inserts into the host's cell membrane). Thus, it will be of great utility to researchers working in this field and foster the development of antiviral therapies targeting fusion proteins.

Together, these pieces of work represent some of the key efforts from the computational community to address the present and future challenges in infectious diseases.

## Author Contributions

All authors contributed to the article and approved the submitted version.

## Conflict of Interest

The authors declare that the research was conducted in the absence of any commercial or financial relationships that could be construed as a potential conflict of interest.

## Publisher's Note

All claims expressed in this article are solely those of the authors and do not necessarily represent those of their affiliated organizations, or those of the publisher, the editors and the reviewers. Any product that may be evaluated in this article, or claim that may be made by its manufacturer, is not guaranteed or endorsed by the publisher.

